# Biochemical and Pathological Studies on Peroxidases –An Updated Review

**DOI:** 10.5539/gjhs.v6n5p87

**Published:** 2014-05-13

**Authors:** Amjad A. Khan, Arshad H. Rahmani, Yousef H. Aldebasi, Salah M. Aly

**Affiliations:** 1 Department of Basic Health Sciences, College of Applied Medical Science, Qassim University, Qassim, Buraidah, Saudi Arabia; 2 Department of Medical Laboratories, College of Applied Medical Science, Qassim University, Qassim, Buraidah, Saudi Arabia; 3 Department of Optometry College of Applied Medical Science, Qassim University, Qassim, Buraidah, Saudi Arabia; 4 Department of Pathology, Faculty of Veterinary Medicine, Suez Canal University, Ismalia, Egypt

**Keywords:** eosinophil peroxidase, glutathione peroxidase, human diseases, lactoperoxidase, myeloperoxidase, oxidative stress, salivary/oral peroxidase, thyroid peroxidase

## Abstract

Peroxidases represent a family of isoenzymes actively involved in oxidizing reactive oxygen species, innate immunity, hormone biosynthesis and pathogenesis of several diseases. Different types of peroxidases have organ, tissues, cellular and sub-cellular level of specificities in their function. Different diseases lead to varied expressions of peroxidases based on several mechanisms proposed. Several researches are going on to understand its deficiency, over-expression and malfunction in relation with different diseases. Some common diseases of mankind like cancer, cardiovascular diseases and diabetes directly or indirectly involve the role of peroxidases. So the status of peroxidase levels may also function as a marker of different diseases. Although many types of diseases in human beings have a strong correlation with tissue specific peroxidases, the clear role of these oxido-reductases is not yet fully understood. Here we are focusing on the role of peroxidases in relations with different diseases occurring due to oxidative stress.

## 1. Introduction

Reactive oxygen species (ROS) are constantly generated in various metabolic activities of all aerobic organisms. These ROS are involved in various normal cellular activities but any imbalance in their production leads to oxidative stress (Burdon, 1995; Droge, 2002). The oxidative stress results in elevated oxyradical generation, protein and other macromolecular oxidation, and finally leads to different diseases (Everse & Coates, 2005).

Recent research shows that controlled level of ROS have biochemical importance as well; as they are used in intracellular signalling, regulate several kinases, transcriptional factors and the cell death machinery (Nomura et al., 2001). ROS have a major role in cytotoxicity and apoptosis (Camandola et al., 2000) and contribute to aging and many human diseases (Lenaz, 1998). These ROS can damage almost all biomolecules if their amount exceeds beyond a normal level (Fridell et al., 2005).

To combat ROS generated complications, cells have developed in parallel a complex enzymatic and non-enzymatic antioxidant defence systems among which peroxidases play an important role (Caremel-Harel & Storz, 2000; Rowinski et al., 2013).

## 2. Peroxidases

Peroxidases belong to a large family of isoenzymes present in almost all living organisms. These are generally heme containing enzymes ranging in Mw from 35-100 Kd (O’Brien, 2000). Mammalian peroxidases are much larger proteins (576-738 amino acids) than the plant counterparts. Peroxidases exist as monomers, dimmers or tetramers and their gene locations also vary among different chromosomes. For example, Glutathione peroxidase 4 (GPx4) is a monomer (gene locus on chromosome 19 p13.3), Eosinophil peroxidase (EPO) exists as a dimer (gene locus on chromosomes 17), while Gluatathione peroxidase 1 (GPx1) is a homotetramer (gene locus on chromosome 3 p21.3) (O’Brien, 2000; Brigelius-Flohe & Maiorino, 2013).

In mammals, peroxidases have some organ, tissue, cellular and sub-cellular specific distribution patterns, performing some specific functions. These peroxidases include, glutathione peroxidase (GPx), myeloperoxidase (MPO), eosinophil peroxidase (EPO), uterine peroxidase, lactoperoxidase (LPO), salivary peroxidase (SPO) and thyroid peroxidase (TPO) (Klebanoff, 2005). These peroxidases play an important role in wide metabolic activities and are directly or indirectly involved in various diseases (Cheng et al., 2008).

Peroxidases generally use H_2_O_2_ as one of the substrates and participate in oxidizing drug and xenobiotic detoxification, innate immunity, hormone biosynthesis and the pathogenesis of inflammatory diseases (Lubos et al., 2011). Even though peroxidases perform a great role in protective mechanisms but still some peroxidases can also lead to some deleterious reactions like co-oxidation of endogenous substrates, drugs and xenobiotics which lead to lipoprotein oxidation, carcinogenesis and liver necrosis. So nowadays even variety of inhibitors are also used against different types of tissue specific peroxidases to treat various types of diseases (Schulz et al., 2012).

### 2.1 Reaction Mechanism of Peroxidases

Generally peroxidases are heme containing oxido-reductases, and after binding substrates undergo series of redox reactions. The heme group of native peroxidase is usually feriprotoporphyrin IX, containing four pyrrole rings to Fe (III). On proximal side, the fifth coordination position is occupied by the imidazole side chain of histidine residue. The sixth coordination is used in the reaction progression (Wirstam et al., 1999). Different human peroxidases have varied substrate specificities, redox properties and kinetics of interconversion of redox intermediates, even though they share similar functional or structural homology (Furtmuller et al., 2006). The majority of the reactions of peroxidases involve interaction with an oxidants such as H_2_O_2_, alkyl hydroperoxides, peroxybenzoic acids, as well as OCl^−^, OBr^−^, ClO_2_^−^, BrO_3_^−^, IO_4_^−^, *m*-nitrobenzoic acid etc.

**Figure 1:**
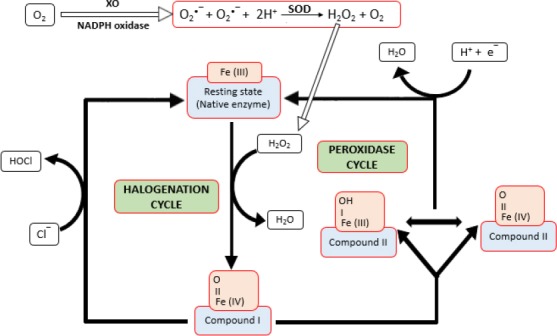
Fundamental steps of the catalytic cycle of heme peroxidases. Free radicals O_2_^•−^ (hydroxyl radical) is generated by the action of xanthine oxidase (XO) or NADPH oxidase and H_2_O_2_ is formed from these free radicals by superoxide dismutase (SOD)

Depending on the substrate availability and the type of peroxidase, these oxido-reductases either go through peroxidase cycle or halogenation cycle (Flemmig et al., 2014) (Figure 1).

Several intermediate forms of peroxidases (compound I and compound II) are formed during these reaction cycle paths (Paumann-Page et al., 2013). Both peroxidase and halogenation cycle starts by reaction of the Fe (III) form of the peroxidase (native enzyme) with hydrogen peroxide to form compound I, which contains two oxidizing equivalents more than the resting enzyme (Wirstam et al., 1999; O’Brien 2000; Kettle et al., 2011).

All heme peroxidases can oxidize halide ions via halogenation cycle through compound I and generally the ease of oxidation of these ions is as: I^−^ > Br^−^ > Cl^−^ (O’Brien, 2000). In peroxidase cycle, compound I is reduced by two successive one-electron step via compound II. In these one-electron oxidation reactions, numerous substrates are oxidized to their corresponding radicals (Furtmuller et al., 2006).

### 2.2 Regulation of Peroxidases

Several diverse factors are responsible for the regulation of peroxidase activity in a cell. Different types of pathogens like bacteria also induce or suppress peroxidase mRNA levels in different organisms (Zhang et al., 2011). Exposure of heavy metals like Cd, Cu, Cr and Ni either activate or inhibit peroxidase level (Woo et al., 2009). Selenium level regulates the GPx mRNA and its enzyme activity as it is a part of selenocysteine, an important amino acid found in glutathione peroxidases.

During different diseases, increased or decreased expressions of peroxidases have been defined by several mechanisms, but these mechanisms do not satisfactorily explain the role of these enzymes. For example, some tumour cells have been observed to have lower GPx activity because tumour cells deplete the level of glutathione (GSH). This GSH depletion stimulates the tyrosinase activity (del Marmol et al., 1993). Tyrosinase activation in melanoma cells leads to higher pigmentation, which is a compensatory mechanism for the decreased GPx levels as melanin is also considered a scavenger of active oxygen species (Ridnour et al., 2004).

One of the characteristics of ROS is role in oxidative DNA mutations and such DNA mutations can be prevented by several peroxidases which counteract the production of proinflammatory mediators such as prostaglandins and leukotrienes (Flohe & Brigelius-Flohe, 2006). So different Inflammatory processes in several diseases like cancer, diabetes, asthma, pneumonia result in up-regulation of some specific peroxidases and prevent initiation phase of these diseases at least.

## 3. Role of Different Peroxidases in Human Diseases

Peroxidases are directly or indirectly correlated with some leading diseases of mankind like Parkinson’s disease, coronary artery disease (CAD) (Tang et al., 2008), convulsive diseases (Kawakami et al., 2006), periodontal diseases (Patel et al., 2009), skin diseases and cancer (Brigelius-Flohe & Kipp, 2009). These diseases can also arise due to several different agents like auto-antibodies, flavonoids and thiocyanates. which involve the metabolic pathways of peroxidase action.

The role of major types of peroxidases in human beings and their correlation with different types of diseases is described here.

### 3.1 Glutathione Peroxidases

Glutathione peroxidases (GPx) are heme thiol peroxidases, comprising a family of eight isoenzymes (GPx1-8) with diverse functions besides catalysing reduction of H_2_O_2_ or organic hydroperoxides to water or alcohols (Ursini et al., 1995). GPx family members have varied distribution in human body between different organs, tissues or cellular compartments (Khan & Gowder, 2014).

GPx1 is the most abundant among GPx family proteins as it is found in erythrocytes and other tissues. It protects these cells from harmful effects of H_2_O_2_ produced by coupled oxidation of different hydrogen donors with oxyhemoglobin (Brigellius-Flohe & Kipp, 2009). Different types of diseases have been reported due to either over or under-expression of GPx1 like hyperglycemia, hyperbilirubinemia and obesity (McClung et al., 2004).

GPx2 is mainly expressed in gastrointestinal tract and it is produced more during squamous cell carcinoma (Serewko et al., 2002), and colorectal cancer (Chiu, 2005). GPx3 has also been found to be expressed more during chemotherapy of head and neck cancer patients (Chen et al., 2011), diabetic and obese subjects (Baez-Duarte et al., 2012). Besides this, down-regulation of this enzyme occurs during endometrial adenocarcinoma (Falck et al., 2010).

GPx4 lowers hydroperoxide level in cells and is responsible for reducing many types of inflammations (Papp et al., 2007), sperm maturation and motility (Kriska et al., 2008). GPx5 has more specific location in epididymis playing a great role in fertility and helps proper embryonic development (Zhang et al., 2008). Little knowledge is still available about the GPx6. GPx7 and GPx8 play a role in protein disulphide isomerization, so involved in folding of different types of proteins (Bosello-Travain et al., 2013).

#### 3.1.1 Regulation of Glutathione Peroxidases Enzyme Level

Selenium level directly regulates the quantity of different forms of GPx proteins. At low selenium level, the GPx1 mRNA degrades faster in cytoplasm as compared to GPx2 and GPx4 mRNA (Brigelius-Flohe and Maiorino, 2013). Selenocysteine, an unusual amino acid is encoded by UGA codon present in GPx mRNA (Chambers et al., 1986). It is usually meant for stop translation for the other proteins, but a new mechanism has been evolved in GPx mRNA, where it codes for Selenocysteine. Sec-tRNA is produced from serine bound to tRNA^(ser)sec^ by selenophosphate synthatase-2 (SPS2), selenophosphate and Sec synthase (Xu et al., 2007).

**Figure 2:**
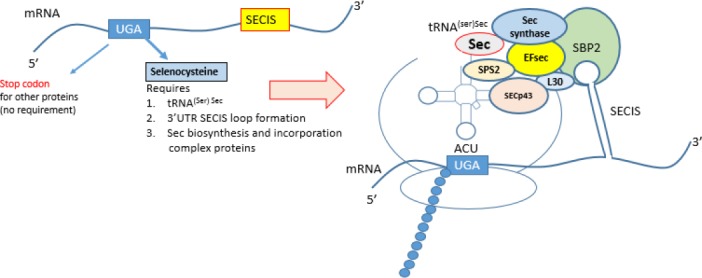
Selenocysteine containing Glutathione peroxidase biosynthesis model

Several macromolecular complexes, proteins and enzymes are involved for this differentiation between stop to Selenocysteine translation signal. These complexes include a specific Selenocysteine containing tRNA (t-RNA^(ser)sec^), the second requirement is a stem loop structure formation in the 3’ untranslated region (3’UTR) of GPx mRNA. This stem loop is called Selenocysteine insertion sequence (SECIS) (Shen et al., 1993). This SECIS recruits a chain of specific tRNA^(ser)sec^ binding proteins and form a complex called Sec incorporation complex. These proteins include SECIS binding protein 2 (SBP2), Sec elongation factor (EF^Sec^), SEC P43, ribosomal L30 and nuclease sensitivity element binding protein 1 (NSEP1) (Chavatte et al., 2005; Shen et al., 2006; Donowan et al., 2008) (Figure 2).

3’UTR is the most important to determine the type of GPx form to be translated. Eukaryotic initiation factor 4a3 (eIF4a3) interacts with a subset of selenoprotein mRNA and prevents binding of SBP2 and thus stops translation (Budiman et al., 2009). This elongation factor (eIF4a3) is induced in selenium deficiency and binds to type-1 SECIS elements, which is SECIS in GPx1 but not in GPx4. So in selenium deficiency, Sec incorporation complex is not formed and GPx1 mRNA does not get translated. Another factor which discriminates between selenoprotein-specific SECIS is nucleolin. Nucleolin has been shown to bind to GPx1 SECIS and thus might link GPx1 mRNA to other proteins of the selenoprotein translation complex (Wu et al., 2000).

### 3.2 Thyroid Peroxidase

Thyroid peroxidase also called as thyroperoxidase (TPO) is mainly expressed in thyroid organs. It is a large transmembrane glycoprotein with covalently linked haem, present in cells on the apical membrane (Gardas et al., 1999). In thyroid glands; iodination of tyrosine for the biosynthesis of thyroid hormone is an important key reaction catalyzed by TPO. TPO also catalyzes the formation of thyroglobulin mono-iodotyrosine and thyroglobulin di-iodotyrosine to form thyroglobulin bound thyroxine (Cheng et al., 2008). A direct correlation has been observed between thyroid disease and TPO (Bakker et al., 2000) as the genetic deficiency of this enzyme causes congenital hypothyroidism (Roos et al., 2010).

Patients with complications of Grave’s hyperthyroidism (Robert & Raeburn, 2009), postpartum thyroiditis (Stagnaro-Green, 2004), and Hashimoto’s thyroiditis (Roberts & Ladenson, 2004) have different levels of autoantibodies present against TPO which represent a hallmark of autoimmune thyroid diseases (Prummel, 2005). Further, in euthyroid subjects, thyrotropin (TSH) is associated with autoantibody (TPOAb) titers (Strieder et al., 2003). Lymphocytic infiltration of TPO antibodies during early stages of development can be seen as predictor for the development of hypothyroidism in future (Scofield, 2004). One of the important complications during pregnancy can be hypothyroidism, so measurement of TPO autoantibodies is recommended strongly. If first trimester of pregnancy shows highest levels of TPO autoantibodies, it can lead to development of hypothyroidism in the postpartum period (Chadha & Goel, 2009).

Thyroid peroxidase is also inactivated by consumption of a number of diets, rich in flavinoids, so such foods can lead to goitre or even thyroid cancer formation. So Infants who receive foods containing excessive soy can show goitre, hypothyroidism and autoimmune thyroid disorders as well (O’Brien, 2000).

### 3.3 Lactoperoxidase

Lactoperoxidase (LPO) is found in wide range of mammalian and human tissues, glands and their secretions. It includes mammary, lachrymal and salivary glands and their secretions like milk, colostrum, tears and saliva (Kussendrager and van Hooijdonk, 2000; Conner et al., 2002; Seifu et al., 2005; Atasever et al., 2013). It contributes to the non-immune host defence system, exerting bacteriostatic and bactericidal activity mainly on Gram negative bacteria (Touch et al., 2004). It also plays an important role against pathogenic microorganisms in intestinal tract of newborn infants (Shin et al., 2000).

Lactoperoxidases have a protective role in respiratory tract. It provides the sterility of different secretions by acting as antibacterial and bacterial clearance agent. For the proper activity of LPO, thiocyanate (SCN^-^) is a requisite component for its active role, so the defects in SCN^-^ transport channels result in loss of LPO activity. This problem can lead to chronic respiratory infections as commonly seen in cystic fibrosis patients. Furthermore, loss of LPO system can increase the chances of *H. pylori*, *Staphylococci*, *E. coli* and *Pseudomonas* infections (Boots & Floris, 2006; Fweja et al., 2008).

LPO has been found in good amount in goat milk and its consumption inhibits the growth of some fungi like *Aspergillus*, *P. chrysogenum*, *T. species*, *phytopthora* and *A. Flavus* (Jacob et al., 2000) but increased expression of LPO activity in milk is a direct indication of mastitis, an inflammation of the breast tissue commonly caused by *S. epidermidis* and *Streptococci*. Further, orally administered LPO ameliorates induced colitis in mice, with symptoms similar to ulcerative colitis in humans (Shin et al., 2008).

Lactoperoxidase present in the brain (brain peroxidase) play an important role in different metabolic events associated with Parkinson’s disease. Role of LPO in Parkinson’s disease is confirmed as the cytotoxic activity of this enzyme is fully inhibited by neuroactive compounds like dopamine, reduced glutathione, and L-cysteine (Everse & Coates, 2004, 2005). Furthermore, LPO present in the skin play an important role in the biosynthesis of melanin in vitro. This function may be relevant to the physiological functions of the melanin pigments in vivo (Garcia-Molina et al., 2005).

### 3.4 Salivary/Oral Peroxidase

Different harmful microorganisms enter the human body through the oral passage regularly. These bacteria are killed by the first line of defence system present in saliva, which includes salivary peroxidase (SPO) as the major peroxidase. Oral peroxidases OPO are composed of salivary peroxidase (80%) and MPO (20%) (Pruitt et al., 1990; Nagler et al., 2002). SPO inhibits both Gram-positive and Gram-negative oral and non-oral bacteria. In addition to this, SPO shows antiviral (Yamamoto et al., 1991; Chase & Klebanove, 1992; Mikola et al., 1995), and antifungal (MacCarthy & Dahl, 1989; Lenander-Lumikari, 1992) activities.

Salivary peroxidase also forms an oral antioxidant system especially against the attack of free radicals produced by cigarette smoke which can lead to oral cancer. It has been observed that smoking even a single cigarette results in a sharp drop of OPO activity. This results in increased carbonylation of the salivary proteins an indication of the oxidative damage to the proteins. Heavy smokers have less OPO activity against the deleterious effects of thiocyanate ions and hydroxyl radicals produced by higher H_2_O_2_. This makes the way easy for the saliva mediated initiation and progression of oral cancer in heavy smokers (Reznick et al., 2003).

### 3.5 Eosinophil Peroxidases

Eosinophil granulocytes or eosinophils are type of white blood cells actively involved in immune system against multicellular parasites and other infections. Eosinophil granules contain a good quantity of eosinophil peroxidase (EPO) (40%) which performs a vast majority of functions during different diseased states. EPO is actively involved in Cl^−^, Br^−^, I^−^ and SCN^−^ oxidation. During infectious state eosinophils are responsible for killing multicellular parasites such as nematode worms involved in filariasis and also certain bacteria such as *M. tuberculosis*. Furthermore, elevation or activation of eosinophils leads to the release of granular proteins linked with variety of inflammatory diseases including allergic diseases of the skin (e.g., atopic dermatitis (Leiferman, 1989)), the lungs (Lacoste et al., 1993) and gastrointestinal tract (e.g., eosinophil esophagitis (Rothenberg et al., 2001)). In addition to this, EPO is also involved in autoimmune neurologic disorders (e.g., multiple sclerosis; (Correale & Fiol, 2004) cancer (Samoszuk, 1997) transplantation rejection and infection with parasitic (Klion & Nutman, 2004) and fungal agents (Schubert, 2006).

Direct evidence of the role of EPO in the pathogenesis is scanty, despite all the above described eosinophil related diseases. Development of EPO knockout mouse line (Denzler et al., 2001) has greatly aided the analysis of the role of EPO in the pathogenesis of different diseases.

### 3.6 Myeloperoxidase

Myeloperoxidase (MPO) is packed inside the cytoplasmic azurophilic granules of neutrophils and is involved in unspecific immune defence system responsible for microbicidal activity (Kajer et al., 2014). MPO catalyzes lipid peroxidation via tyrosyl radical formation (Savenkova et al., 1994) and this leads to generation of other products which cause lipoprotein oxidation (Daugherty et al., 1994). Oxidation of lipoproteins like HDL contributes to atherosclerosis by counteracting HDL anti-atherogenic effects (Bergt et al., 2004; Zheng et al., 2004).

MPO has been strongly implicated in other disease like rheumatoid arthritis, atherosclerosis and lung cancer (Hoy et al., 2002). It has been reported that some chest pain patients show significant MPO levels and MPO oxidation products have been observed in brains of patients diagnosed with Alzheimer’s disease and multiple sclerosis (Nagra et al., 1997; Reynolds et al., 1999; Crawford et al., 2000; Green et al., 2004). MPO is also released from polymorphonuclear neutrophils and monocytes in acute coronary syndrome after activation and so listed as risk marker in such diseases (Mocatta et al., 2007; Ndrepepa et al., 2008).

Recent research shows that MPO is an emerging biomarker to assess cardiovascular diseases (CVD) and endothelial dysfunction in vivo (Eiserich et al., 2002; Brennan & Hazen, 2003) as human subjects with significantly lower risks of CVD were found to have less MPO activity and *vice-versa* (Zhang et al., 2001). Even though this enzyme has been used as a risk marker in this syndrome (Morrow et al., 2007), its proper role in such patients is not yet well defined. There is also a strong association between carotid atherosclerosis and the MPO in patients whose HDL cholesterol levels are less than desirable value (Exner et al., 2006).

It has also been observed that inherited MPO defects can lead to impaired fungicidal activity which can lead to candidiasis (Cheng et al., 2008). Furthermore, MPO leads to the development of atheroma and plaque rupture as it generates reactive oxidants and radicals as well.

## 4. Other Peroxidases

Several other peroxidases and related diseases have been identified, but are less characterized. These peroxidases have also some tissue specific distribution and functions. These include vascular peroxidase, uterine peroxidase, prostaglandin H1/2 synthase, etc. Uterine peroxidase plays an important role in oestrogen-induced uterine hyperaemia and uterine weight by conversion of oestrogen to their catechol forms (Farley et al., 1992). Several eye diseases like cataract and macular degeneration may be related with the oxidative mechanisms also, as few studies have shown that high levels of GPx are associated with age related macular degeneration (Delcourt et al., 1999). These peroxidases have more or less common mechanism of action with other common peroxidases and promising research is going on in understanding their proper roles.

## 5. Conclusion

Different types of oxyradicals once considered as harmful products are now know to perform some essential cellular functions. But any imbalance in their production leads to varied diseases, so putting forward the burden of this stress on peroxidases as well. Peroxidases play a significant role in antioxidant defense system of living organisms and are actively involved in oxyradical oxidation, hormone biosynthesis, and innate immunity. Different peroxidases have organ, tissue, cellular or sub-cellular specificities and are directly or indirectly involved in various diseases of mankind. During different diseases, the expression of peroxidases either increases or decreases. Several mechanisms have been suggested to explain their varied expressions during diseased states. Even though peroxidases have been used as risk markers in different human diseases but its perfect role is not yet well defined.
